# Functional Analysis of a Complement Polymorphism (rs17611) Associated with Rheumatoid Arthritis

**DOI:** 10.4049/jimmunol.1402956

**Published:** 2015-02-27

**Authors:** Joanna L. Giles, Ernest Choy, Carmen van den Berg, B. Paul Morgan, Claire L. Harris

**Affiliations:** * Institute of Infection and Immunity, School of Medicine, Cardiff University, Cardiff CF14 4XN, United Kingdom;; † Cardiff Regional Experimental Arthritis Treatment and Evaluation Centre, Section of Rheumatology, School of Medicine, Cardiff University, Cardiff CF14 4XN, United Kingdom; and; ‡ Institute of Molecular and Experimental Medicine, School of Medicine, Cardiff University, Cardiff CF14 4XN, United Kingdom

## Abstract

Complement is implicated in the pathogenesis of rheumatoid arthritis (RA); elevated levels of complement activation products have been measured in plasma, synovial fluid, and synovial tissues of patients. Complement polymorphisms are associated with RA in genome-wide association studies. Coding-region polymorphisms may directly impact protein activity; indeed, we have shown that complement polymorphisms affecting a single amino acid change cause subtle changes in individual component function that in combination have dramatic effects on complement activity and disease risk. In this study, we explore the functional consequences of a single nucleotide polymorphism (SNP) (rs17611) encoding a V802I polymorphism in C5 and propose a mechanism for its link to RA pathology. Plasma levels of C5, C5a, and terminal complement complex were measured in healthy and RA donors and correlated to rs17611 polymorphic status. Impact of the SNP on C5 functionality was assessed. Plasma C5a levels were significantly increased and C5 levels significantly lower with higher copy number of the RA risk allele for rs17611, suggesting increased turnover of C5 V802. Functional assays using purified C5 variants revealed no significant differences in lytic activity, suggesting that increased C5 V802 turnover was not mediated by complement convertase enzymes. C5 is also cleaved in vivo by proteases; the C5 V802 variant was more sensitive to cleavage with elastase and the “C5a” generated was biologically active. We hypothesize that this SNP in C5 alters the rate at which elastase generates active C5a in rheumatoid joints, hence recruiting neutrophils to the site thus maintaining a state of inflammation in arthritic joints.

## Introduction

Rheumatoid arthritis (RA) is a chronic inflammatory disease affecting almost 1% of the population worldwide (1). RA susceptibility involves a combination of genetic factors and environmental triggers such as infection and smoking (2). Genome-wide association studies identified multiple genetic links to RA (3–6); several implicated the TNFR-associated factor (TRAF)1/complement C5 region as a susceptibility locus.

Complement is a key component of innate immunity with roles in protecting against infection and linking innate and adaptive immune responses. Complement is a proteolytic cascade comprising more than 30 proteins; the activation pathways (classical, alternative, and lectin) involve sequential events, generating new enzymes and active fragments (7–9). Cleavage of C3 generates anaphylactic (C3a) and opsonic (C3b) fragments. Subsequent cleavage of C5 yields the anaphylactic fragment C5a and C5b which initiates assembly of the lytic membrane attack complex.

There are strong links between the pathogenesis of RA and the complement system (10). Complement activation fragments are elevated in plasma and synovial fluid in patients (11, 12), and complement activation products are found in joint tissues leading to the suggestion that joint damage is in part mediated by complement (13). Evidence from experimental models also implicates complement in RA; anti-C5 Ab ameliorated disease in mouse collagen-induced arthritis in mice-ameliorated disease (14, 15), and C5-deficient mice were refractory to collagen-induced arthritis (16).

Complement polymorphisms influence risk in many diseases (17); small differences in activities of polymorphic variants, when inherited in some combinations (the complotype), dramatically alter complement activity and impact disease risk (17, 18). TRAF1/C5 is a highly polymorphic locus on chromosome 9 linked to RA susceptibility (19, 20). Many of the susceptibility single nucleotide polymorphisms (SNPs) are in linkage disequilibrium with each other. We focused on a coding SNP in this locus linked to RA in multiple studies, rs17611, a miss-sense polymorphism causing a V802I change in C5 (4, 20). The SNP is also linked to periodontitis (21), poor outcome in pneumococcal meningitis (22), adverse cardiovascular outcomes (23), and stroke risk (24). No functional basis for these disease associations has been described.

In this study, we show that individuals homozygously expressing the RA risk s17611 allele exhibit increased C5a and decreased C5 in plasma, evidence of increased C5 turnover. We demonstrate that C5_V802I_ variants are indistinguishable in complement activation assays; however, the risk C5_V802_ variant is more susceptible to cleavage by human neutrophil elastase (HNE), generating an active fragment functionally indistinguishable from C5a. We suggest that increased susceptibility to cleavage by HNE, with release of proinflammatory fragments, explains the association of the polymorphism with RA and other inflammatory diseases in which elastase levels are locally elevated.

## Materials and Methods

### DNA preparation and genotyping

Blood was collected into EDTA from healthy donors and from RA and osteoarthritis patients satisfying American College of Rheumatology/European League Against Rheumatism classification criteria (25). An aliquot was used for gDNA preparation and plasma harvested from the residual stored at −80°C. gDNA was purified using a RELIAprep gDNA kit (Promega, Southampton, U.K.) following manufacturer’s instructions. gDNA concentration was determined using a Nanodrop 1000 spectrophotometer (Thermofisher, Loughborough, U.K.). All samples were normalized to 10 ng/ml and genotyped at the rs17611 SNP using Kaspar technology (Kbiosciences, Teddington, U.K.).

### Measurement of complement components

C5, C5a, C3a, and the terminal complement complex (TCC) were measured in EDTA plasma from genotyped donors. C5a and C3a were measured using commercial ELISA kits (Microvue; Quidel, San Diego, CA), according to the manufacturer’s instructions. TCC levels were measured in an in-house ELISA. Briefly, 96-well Maxisorp plates (Thermofisher) were coated with anti-TCC Ab (aE11, Hycult, The Netherlands) at 2 μg/ml in carbonate/bicarbonate buffer (pH 9.6); plates were blocked in 2% BSA and then incubated with plasma samples. Detection was with HRP-conjugated anti-C8 mAb (clone E2) in 2% BSA and color developed using orthophenylenediamine (Sigma-Aldrich, Dorset, U.K.). Plasma C5 levels were measured using a novel in-house C5 ELISA. Briefly, 96-well Maxisorp plates were coated with an in-house polyclonal rabbit anti-human C5 Ab (8 μg/ml IgG in bicarbonate buffer [pH 9.6]), blocked in 2% BSA, and then incubated with plasma samples diluted 1 in 600 in 2% BSA. Bound C5 was detected using a mouse monoclonal anti-human C5 (MBI-C5-3; developed in-house, 5 μg/ml), followed by donkey anti-mouse IgG HRP (Jackson Labs, Stratech, Newmarket, U.K.; 1 in 2500) and color developed using orthophenylenediamine. Plasma levels of complement components were calculated using Graphpad prism 3 (GraphPad, La Jolla, CA), and statistical analysis was performed using one-way ANOVA. For C5, *p* = 0.0047; for C5a, *p* < 0.0001; and for TCC, *p* = 0.6032.

### C5 purification

Donors homozygous for the C5_V802I_ polymorphic variants were identified and EDTA plasma collected. C5 was purified by affinity chromatography on monoclonal anti-human C5 (MBI-C5-2; 30 mg) immobilized to a 5 ml HiTrap column (GE Healthcare, Fisher Scientific, Loughborough, UK.). on an Akta Purifier system (GE Healthcare). Plasma (50 ml) was loaded onto the column which was then washed in HEPES buffered saline (HBS; 10 mM HEPES and 150 mM NaCl [pH 7.4]; 10-column volumes) and eluted using 2 M MgCl_2_ in HBS. The C5 peak was collected, loaded immediately onto a SD200 gel filtration column (GE Healthcare), and eluted in HBS to remove aggregates and desalt. Purity was confirmed by SDS PAGE and protein concentration measured using the Bradford assay (Bio-Rad) and Nanodrop.

### Add-back hemolytic assays

Functional differences between the two C5 variants were probed using add-back hemolytic assays. C5 was depleted from normal human serum (NHS) by passage over the C5 affinity column equilibrated in ice-cold HBS. Ab-sensitized sheep erythrocytes (ShEA) were prepared using standard protocols (26). C5 was titrated as indicated in 50 μl complement fixation diluent (CFD; Sigma-Aldrich) in a 96-well plate; 50 μl ShEA in CFD was added to the wells, and lysis was developed by adding 50 μl C5-depleted serum diluted 1 in 5 in CFD. To measure rate of lysis, plates were incubated in a 37°C chamber of an Anthos ht3 plate reader (LBMG Labtech, Aylesbury, U.K.), where residual intact cells were measured densitometrically by reading A690 at 3-min intervals for 30 min. The percentage of intact cells was calculated at each time point using the Equation 100*(A690 test sample − A690 100% control)/(A690 0% control − A690 100% control), and rate of lysis plotted using Graphpad prism 3. To calculate end-point lysis, plates were incubated at 37°C for a set time, cells were pelleted by centrifugation, and hemoglobin release was measured by absorbance at 415 nm. Control incubations included 0% lysis (buffer only) and 100% lysis (0.1% Nonidet-P40). Percentage lysis (100 × [A415 test sample − A415 0% control]/[A415 100% control − A415 0% control]) was calculated.

### C5 convertase/terminal pathway hemolysis assays

ShEA-C3b intermediates were generated by incubating ShEA with NHS depleted of factor B and factor H (NHSΔBH) (27); cells were washed, resuspended to 2% in alternative pathway buffer (veronal-buffered saline containing 7 mM MgCl_2_ and 10 mM EGTA). To form C5 convertase, ShEA-C3b were washed, resuspended in alternative pathway buffer, and incubated with factor B [4.09 μg/ml; in-house (28)] and factor D (0.25 μg/ml; Comptech) for 15 min at 37°C. C5 convertase–coated cells were immediately added to titrated doses of C5 and constant C6 (physiological concentration of 65 μg/ml) and incubated (37°C, 10 min) in a 96-well plate. Lysis was developed by adding C7, C8, and C9 (all purified in house; 55, 55, and 60 μg/ml respectively). Rate of lysis and end-point lysis were measured as described above.

### Cobra venom factor assays

Cobra venom factor (CVF) was purified from lyophilised *Naja naja kaouthia* venom (Sigma-Aldrich). Briefly, 200 mg lyophilized venom was solubilized in 10 ml TBS (pH 8.5). Insoluble material was removed by centrifugation, and filtered supernatant was applied to a DEAE sepharose column at 4°C on an Akta Purifier. CVF was eluted using a linear gradient to 1 M NaCl in TBS. Fractions were screened on 7.5% SDS-PAGE gels. Fractions corresponding to CVF were dialyzed into PBS (10 mM phosphate and 150mM NaCl [pH 7.5]) and applied to a SD200 gel filtration column in PBS.

C5 convertase (CVFBb) was prepared by incubating 10 μg CVF, 10 μg factor B, 0.12 μg factor D, and 5 mM MgCl_2_ in 50 μl TBS for 1 h at 37°C as described previously (29). Each C5 variant (30μg) was incubated with 8 μl of the preformed convertase at 37°C. Aliquots (5 μg protein in a 5-μl volume) were removed from the mix at intervals into an equal volume of SDS reducing loading buffer. Samples were run on an 8.5% gel and visualized by Coomassie staining using standard protocols. Band densitometry was measured using ImageJ (http://rsb.info.nih.gov/ij/).

### Enzyme cleavage assays

Each C5 variant (10 μg) was incubated with 0.5 μg HNE (Sigma-Aldrich) in HBS buffer at 37°C. Aliquots were withdrawn at intervals into SDS loading buffer (±DTT) and heated at 100°C for 5 min before running on a 4–12% SDS gradient gel (Invitrogen), stained with Coomassie blue. C5a was detected in HNE digests by Western blot; proteins were transferred from SDS-PAGE to nitrocellulose then probed with anti-C5a (mAb557, Hycult). C5a release was also measured using a C5a ELISA kit as described above (Quidell).

### Calcium Flux assays

U937 cells, stably transfected with the C5aR1 or IL-8RB (a gift from E. Prossnitz, Albuquerque, NM) at 10^7^cells/ml were loaded with 2 μM Fura-2-AM for 30 min at room temperature. Cells were washed and resuspended in Krebs/HEPES/BSA buffer (25 mM HEPES, 120 mM NaCl, 4.8 mM KCl, 1.2 mM KH_2_PO_4_, 1.2 mM MgSO_4_, 1.3 mM CaCl_2_ [pH 7.4], and 0.1% BSA). Cells (200 μl at 5 × 10^6^/ml) were stimulated with C5a, C5, or C5 preincubated with HNE (2 h at 37°C as described above). Changes in intracellular calcium were measured as described previously (30, 31). To confirm that the observed signal was C5a induced, a C5aR1 antagonist peptide (H-Phe-Lys-Pro-D-Cha-Trp-D-Arg-OH) (100 nM diluted in Krebs/HEPES/BSA) was added to the cells 10 min before stimulation with C5-derived cleavage products.

## Results

### Measurement of C5 and C5 activation products in plasma samples

**FIGURE 1.**
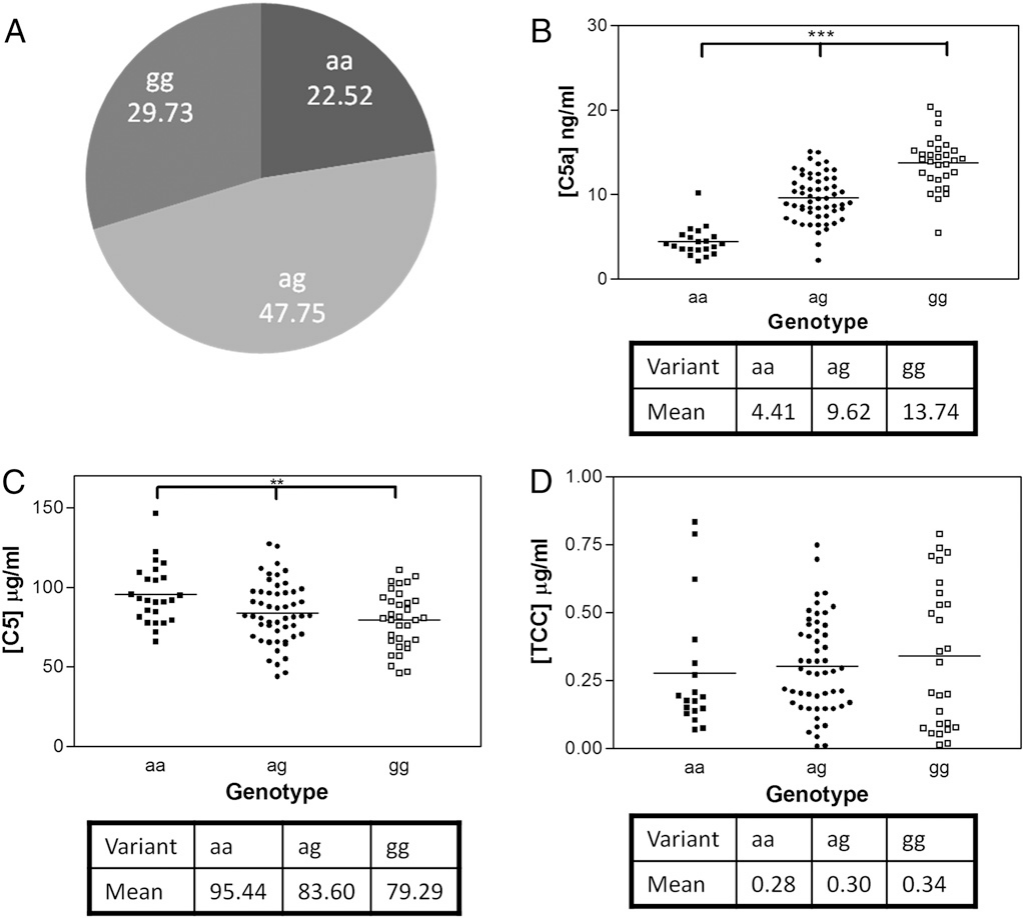
C5 and C5 activation products in plasma samples correlate to rs17611 genotype. (**A**) Genotyping a healthy population for rs17611. The gene frequency of the risk allele (g) in this population is 0.54. (**B**) and (**C**), respectively, show C5a and C5 plasma levels in genotyped individuals measured by ELISA. Plasma levels were observed to be significantly different with increasing copy number the risk variant (g) correlating to higher levels of C5a (***p < 0.001) and lower levels of C5 (**p = 0.05). (**D**) TCC plasma levels were also measured by ELISA and show no significant trend with genotype in these assays.

In 111 healthy donors genotyped for the rs17611 SNP, the minor allele frequency was 0.46, in line with published frequency (National Center for Biotechnology Information reported minor allele frequency 0.41). Plasma levels of C5a, C5, and TCC were measured in the cohort; mean levels of these analytes were comparable to published levels: [C5a] = 9.23 ng/ml, [C5] = 86.1 μg/ml, and [TCC] = 0.31 μg/ml. When the cohort was divided on the basis of rs17611 SNP status, risk allele number was positively associated with [C5a] (*p* < 0.001 by ANOVA) and negatively associated with [C5] (*p* = 0.005) (Fig. 1). Plasma [TCC] was not significantly different between the groups. Attempts to detect C5a in patient and control plasma by Western blotting were unsuccessful, likely because of its very low concentration. Synovial fluid was not available from patients in the cohorts studied.

In a second cohort comprising 80 RA patients, [C5a] again showed strong positive correlation with risk allele number (*p* < 0.001), and [C5] showed a trend to negative correlation that did not reach significance; as in healthy subjects (Supplemental Fig. 1), [TCC] was not different between the groups (data not shown).

To assess whether the observed differences in C5 and C5a concentrations were a result of systemic complement activation, C3a levels were measured in 71 RA patients. There was no significant difference between the groups (Supplemental Fig. 1), leading us to conclude that the increased C5 turnover in risk allele carriers was not due to increased complement activation.

### Complement lytic activity of the two C5 V802I protein variants

**FIGURE 2.**
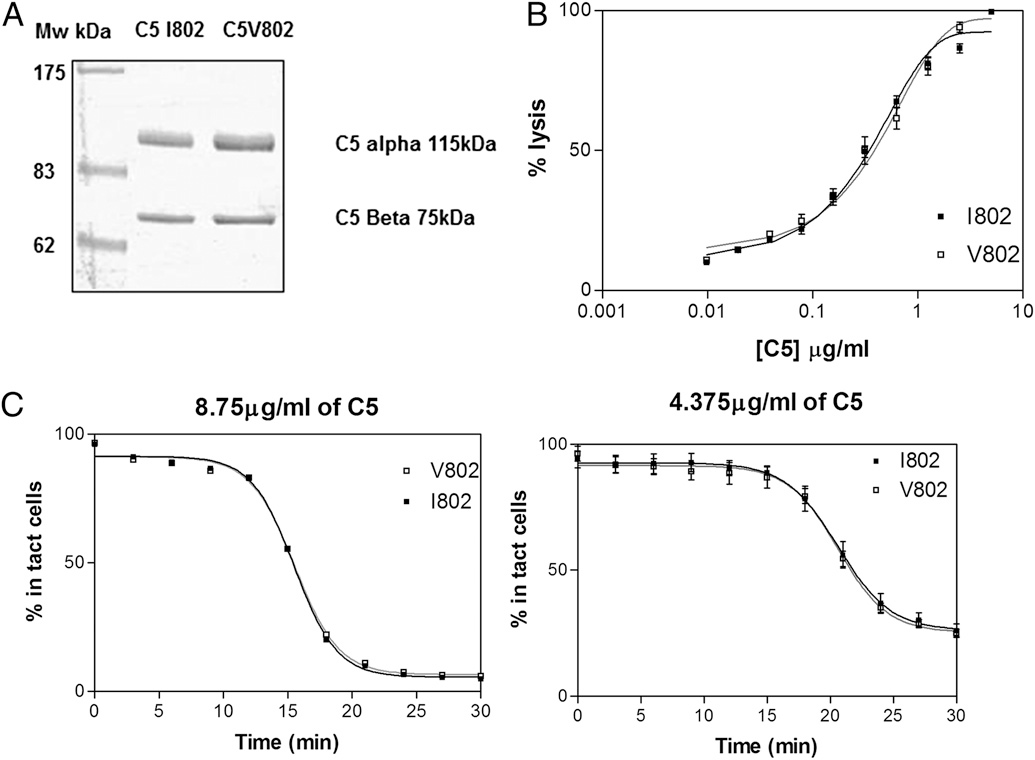
Both C5 V802I variants show comparable complement lytic activity in standard hemolysis assays. (**A**) Two C5 variants were purified by affinity chromatography and subsequent gel filtration. C5 variants were run on a reduced gel to confirm purity. Two bands were present, representing C5 α (115 kDa) and C5 β (75 kDa). (**B**) C5 variants were titrated into C5-depleted serum and ShEA. C6–C9 was then added, and RBC lysis was measured by hemoglobin release after 30-min incubation. Representative results from three experiments are shown. (**C**) The rate of lysis was calculated by measuring the number of intact cells present at 3-min intervals at each concentration of C5 for the C5 add-back assays described. Results were calculated for each concentration in of C5 in (B). The rate curves for two concentrations of C5 are shown. Representative results from three experiments are shown.

To determine whether the decreased C5 and elevated C5a levels in individuals expressing rs17611 risk alleles was the result of higher turnover by the C5 convertase enzyme, hemolytic assays were performed. C5 purified from healthy individuals homozygous for each variant (C5_V802_ or C5_I802_) was titrated back into NHS depleted of C5 (NHSΔC5) and used in hemolysis assays. Neither end point nor rate hemolytic assays showed any significant difference between the C5 variants (Fig. 2).

**FIGURE 3.**
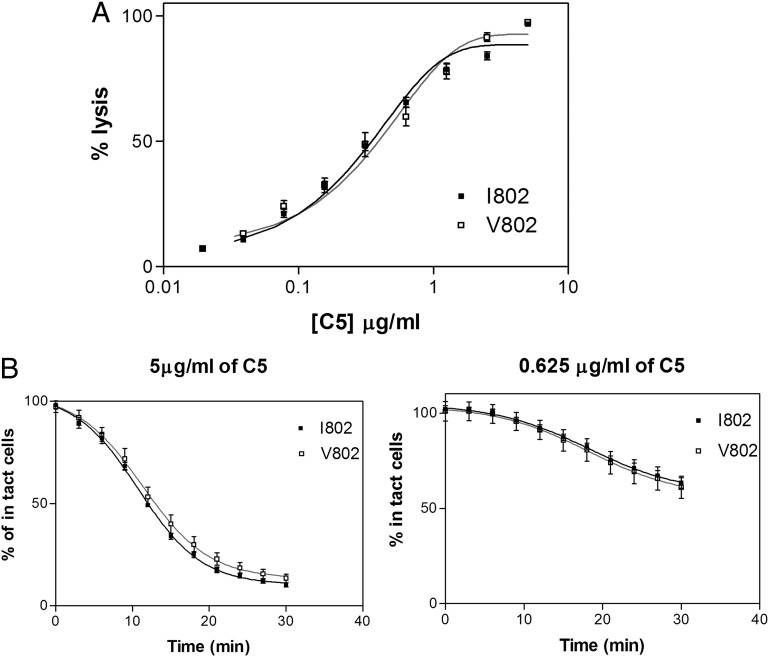
Both C5 V802I variants show comparable complement lytic activity. (**A**) Hemolysis was measured by first coating sheep erythrocytes with C3bBb and then titrating C5 and inducing lysis by adding remaining complement components. The end-point assay is shown after 30-min incubation. Percentage lysis was calculated by measuring hemoglobin release for each C5 concentration at end point. Representative results from more than six experiments are shown. (**B**) The rate of lysis was plotted by measuring absorbance of intact cells at 3-min intervals for each concentration of the C5 add back assays described. Two representative concentrations of C5 are shown. Representative results from six experiments are shown.

To increase the sensitivity of this assay and reveal small differences in C5 function, C3/C5 convertase coated sheep erythrocytes were generated and C5 variant proteins were titrated into the cells. Lysis was developed by addition of purified terminal components. No significant difference in the development of lysis was seen between the two C5 variants (Fig. 3).

Finally, we compared the C5 variants in a CVF C3/C5 convertase assay; no significant difference in rate or extent of cleavage of the variants was observed, either by SDS-PAGE or by C5a ELISA (Supplemental Fig. 2).

These data confirm that the C5_V802I_ variants are not differentially processed by the C5 convertase and provoked us to look for alternative explanations for differences in turnover.

### HNE cleaves C5_V802I_ variants at different rates to produce an active C5a-like fragment

**FIGURE 4.**
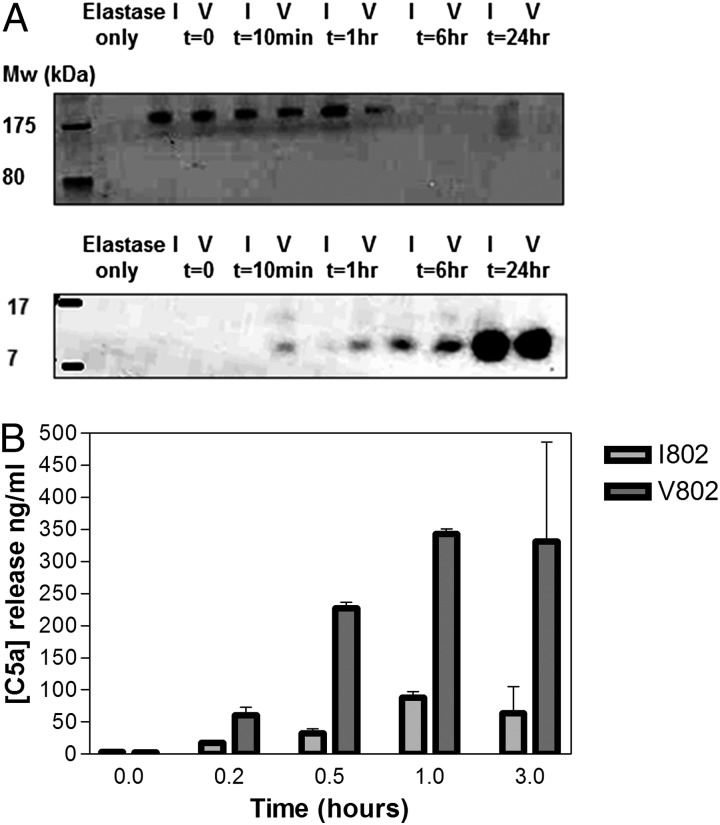
HNE cleaves C5 V802I variants at different rates. (**A**) C5 (I802 and V802) variants were incubated with HNE for 24 h. The reaction was stopped at various time points, and samples were run on a gel (*top gel*) and Western blot (*bottom gel*) and probed for C5a. A C5a-like molecule appeared over time. (**B**) C5 (I802 and V802) variants were incubated with HNE for 3 h. The reaction was stopped at various time points, and samples were measured for the presence of C5a by ELISA.

HNE, an enzyme released from activated neutrophils at inflammatory sites, has been shown to cleave C5 (32), but the resulting fragments have not been fully characterized. To assess the impact of the C5_V802I_ polymorphism on HNE-mediated cleavage, purified C5 variants were digested with HNE. Cleavage of the C5 chains was assessed on SDS-PAGE and appearance of the C5a-like fragment measured by Western blotting with a C5a-specific mAb (Fig. 4A). Both rate of disappearance of intact C5 and appearance of the C5a-like fragment were greater for the C5 risk variant C5_V802_. To confirm this accelerated cleavage, generation of the C5a-like fragment was measured by ELISA; levels were higher for C5_V802_ at all time points in the experiment, up to 6-fold at 60 min (Fig. 4B).

**FIGURE 5.**
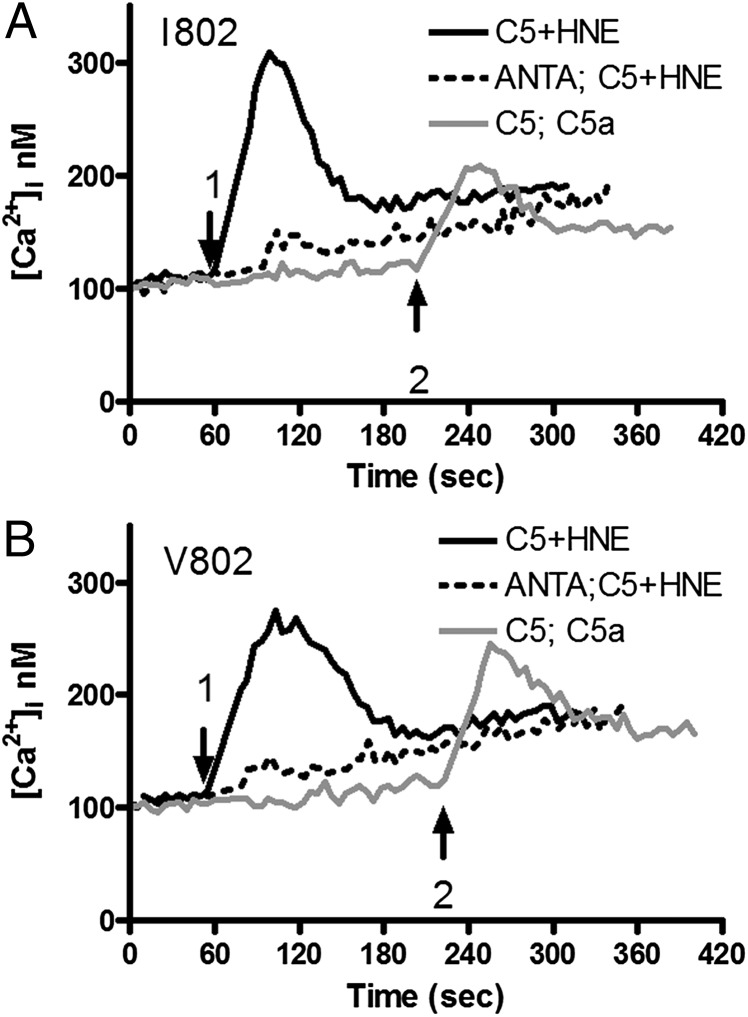
Both C5 variants are functionally active when tested in calcium flux assays. C5aR1-transfected cells were loaded with Fura-2-AM and stimulated with C5 variants I801 (**A**) or V802 (**B**) after incubation with HNE (solid black line) or buffer alone (not shown). Cells stimulated with C5 not incubated with HNE were subsequently stimulated with C5a (0.8 nM). Some cells were preincubated with C5aR antagonist (ANTA) and then stimulated with HNE-incubated C5 (dotted line). Arrow 1, addition of C5 ± HNE; arrow 2, addition of C5a.

The HNE-generated C5a-like fragment migrated at a slightly higher molecular mass compared with recombinant C5a and C5a generated through cleavage of C5 by the CVFBb convertase, as shown by Western blot with a C5a-specific mAb (Supplemental Fig. 3). To test whether this HNE-generated fragment retained C5a activities, C5 was incubated with or without HNE for 2 h and then added to C5aR1-transfected U937 cells preloaded with Fura2-AM. HNE-digested C5 triggered a prompt Ca^2+^ flux, whereas addition of uncleaved C5 or HNE alone had no effect. As a negative control, U937 cells transfected with an IL-8R did not respond to HNE-digested C5 (data not shown). Preincubation with a C5aR1 antagonist blocked the HNE treated C5-triggered increase in intracellular Ca^2+^ (Fig. 5). rC5a caused a Ca^2+^ flux in C5aR1 expressing U937 comparable to that caused by HNE-digested C5 (Fig. 5).

## Discussion

Complement is a key component of the innate immune response, and complement polymorphisms and mutations influence risk in a range of diseases (17). Some complement polymorphisms have been shown to influence complement activity, and the complotype describes the inherited set of complement variants that dictate complement function in an individual (18). Several polymorphisms in C5 have been associated with RA in various populations (4, 20). Multiple SNPs at the C5-TRAF locus have a significant association (e.g., rs3761847 is the most significant with *p* = 2 × 10^−8^), and many of these are in linkage disequilibrium, making it difficult to ascribe causality (20). In this paper, we report that the RA risk allele of a coding SNP in C5 (rs17611) is associated with increased C5 turnover and elevated plasma levels of the potent anaphylatoxin C5a in healthy donors and RA patients. TCC levels did not reflect this difference, likely because of the known inefficiency of TCC generation in the fluid phase (33, 34). C3a levels did not correlate with RA risk allele, suggesting that increased C5 turnover and C5a production occurred independently of downstream complement activation.

To investigate the cause of these differences, functional assays comparing the C5_V802I_ variants were performed. In multiple hemolytic assays, no differences between the C5 variants was observed, indicating that increased turnover of C5_V802_ was not caused by increased susceptibility to cleavage by C5 convertase. These findings eliminate the possibility that increased C5 turnover in normal individuals expressing the risk variant is due to increased complement enzyme cleavage during tickover activation and suggest that other mechanisms of C5 cleavage and C5a generation are responsible.

HNE, a neutrophil-derived enzyme, was previously shown to cleave C5 and, in the presence of C6, generate a C5b6-like hemolytic complex (32). In this study, we showed that C5_V802_, risk for RA, was more readily cleaved by HNE than C5_I802_. These findings suggest that the observed difference in turnover between the C5 variants lies in their susceptibility to cleavage by HNE and perhaps other noncomplement enzymes. It is compelling that this difference is evident even in normal individuals even though HNE plasma levels are normally low and most are bound in an inactive form to α1-protease inhibitor (36); however, during inflammation, neutrophils activated locally and/or systemically release HNE leading to markedly increased local levels (35). HNE cleaves C5 at a site close to but distinct from the C5a cleavage site [after Arg^751^ in the α-chain (32)]; the precise cleavage site is not identified. We noted that the HNE-released fragment was ∼1 kDa larger than convertase-generated C5a (Supplemental Fig. 3); knowledge that HNE preferentially cleaves after small side-chain aliphatic amino acids with Val preferred leads us to predict that the HNE cleavage site in C5 follows Val^760^, nine residues downstream of the C5a terminus at Arg^751^, generating a fragment with a predicted molecular mass of 9307 Da (921 Da larger than C5a). Efforts to confirm this prediction by MALDI-TOF analysis are ongoing. The HNE-generated C5a-like fragment was capable of binding C5aR on indicator cells and triggering Ca^2+^ flux similar to that generated by recombinant C5a, confirming that it was biologically active. It is possible that this novel C5a-like molecule will be more stable than C5a in vivo as it will not be inactivated by des-Argination. We have shown that plasma levels of C5a and C5 are affected by C5_V802I_ status, likely a consequence of increased susceptibility of the C5_V802_ variant to HNE cleavage. In RA, the inflamed joint is full of activated neutrophils and other inflammatory cells. HNE and other proteases are abundant in RA synovial fluid (36, 37); this would lead to local cleavage of C5 and generation of functional C5a-like fragments that in turn drive more inflammation, a process that is enhanced for C5_V802_ and explains its association with inflammatory diseases. Our findings build the case for use of HNE inhibitors in the treatment of RA. HNE has long been implicated in chronic inflammatory disease (38, 39); however, although a number of effective agents have been developed for treatment of other conditions, few have been tested in RA. Sivelestat, a small molecule inhibitor of HNE used in therapy of acute respiratory failure in sepsis (40), already has proved effective in the collagen-induced arthritis model (41). The anti-C5 mAb eculizumab was tested in RA but was not progressed; data on the Alexion Web site shows that although the primary end point was missed, a positive response was achieved at 3 mo in patients stratified according to baseline TCC despite the low dose (8 mg/kg) used. Trials of the C5aR antagonist PMX53 also failed, but this agent has failed in all trials likely because of limited bioavailability (42). Our data suggest that effective inhibition of C5a/C5aR would be therapeutic in RA.

## Supplementary Material

Data Supplement
