# Supplementary material for: Functional Analysis of a Complement Polymorphism (rs17611) Associated with Rheumatoid Arthritis
Source: J Immunol. 2015 Feb 27;194(7):3029–34. doi: 10.4049/jimmunol.1402956 (PMC4367161; doi:10.4049/jimmunol.1402956)
Supplement: Data Supplement [file JI_1402956.zip › JI_1402956_Supplemental_Figures_1.pdf]

A

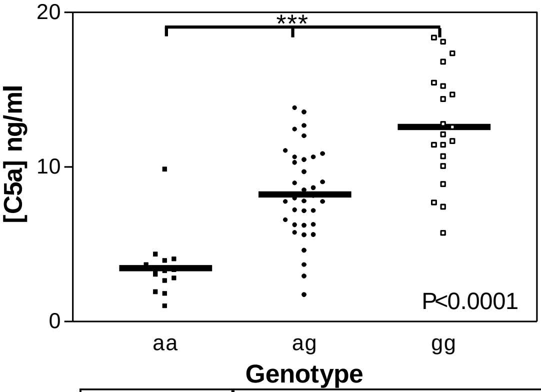

Variant	aa	ag	gg
Mean	3.456	8.221	12.59

B

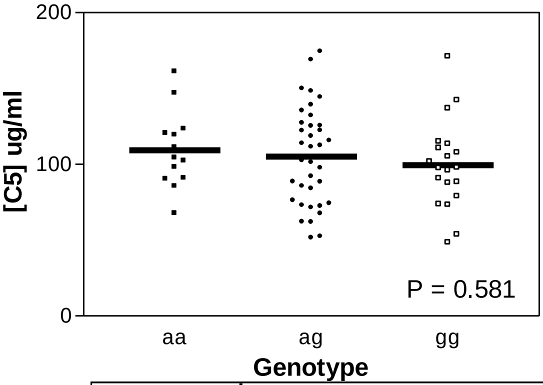

Variant	aa	ag	gg
Mean	109.3	105.1	99.35

C

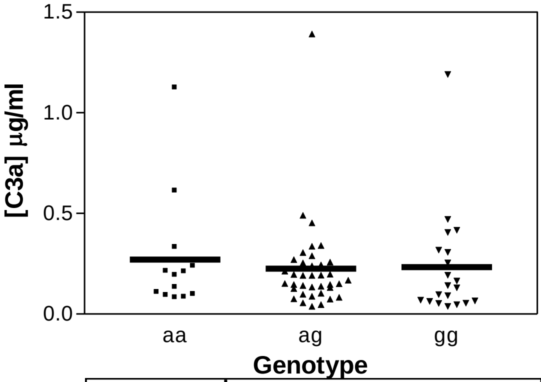

Variant	aa	ag	gg
Mean	0.2704	0.2256	0.2336

Figure S1: **C5 and C5 activation products in plasma samples of RA patients also correlate to rs17611 genotype, C3a levels do not.**

A) B) and C) respectively show C5a, C5 and C3a plasma levels of genotyped RA individuals. Levels were measured by ELISA and were observed to be significantly different between homozygous individuals for C5a, with C5a levels increasing according to copy number of the risk variant (g). C5 levels show the reverse trend, but with no significance owing to limited sample size. C3a levels show no trend and were not significantly different.

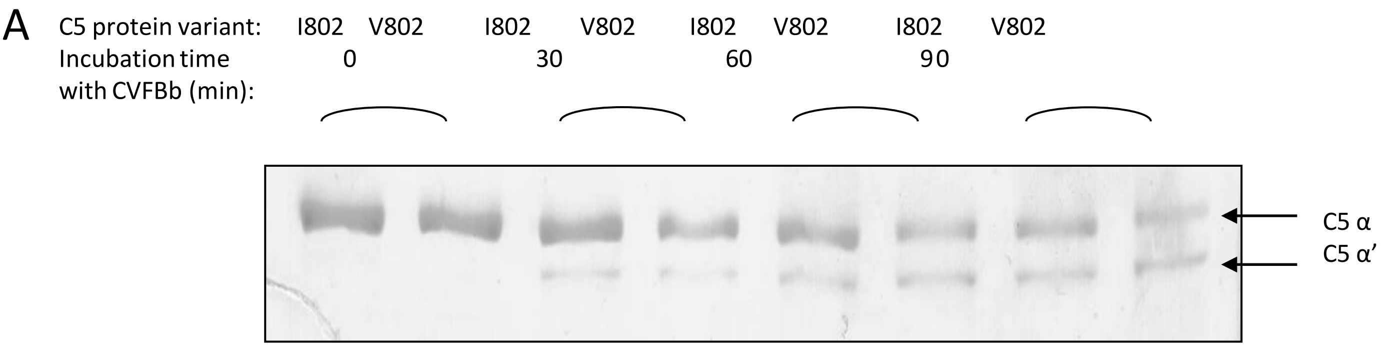

% of C5 cleaved to C5α' measured by densitometry	I802	V802	I802	V802	I802	V802	I802	V802
	0	0	24	27	29	40	38	42

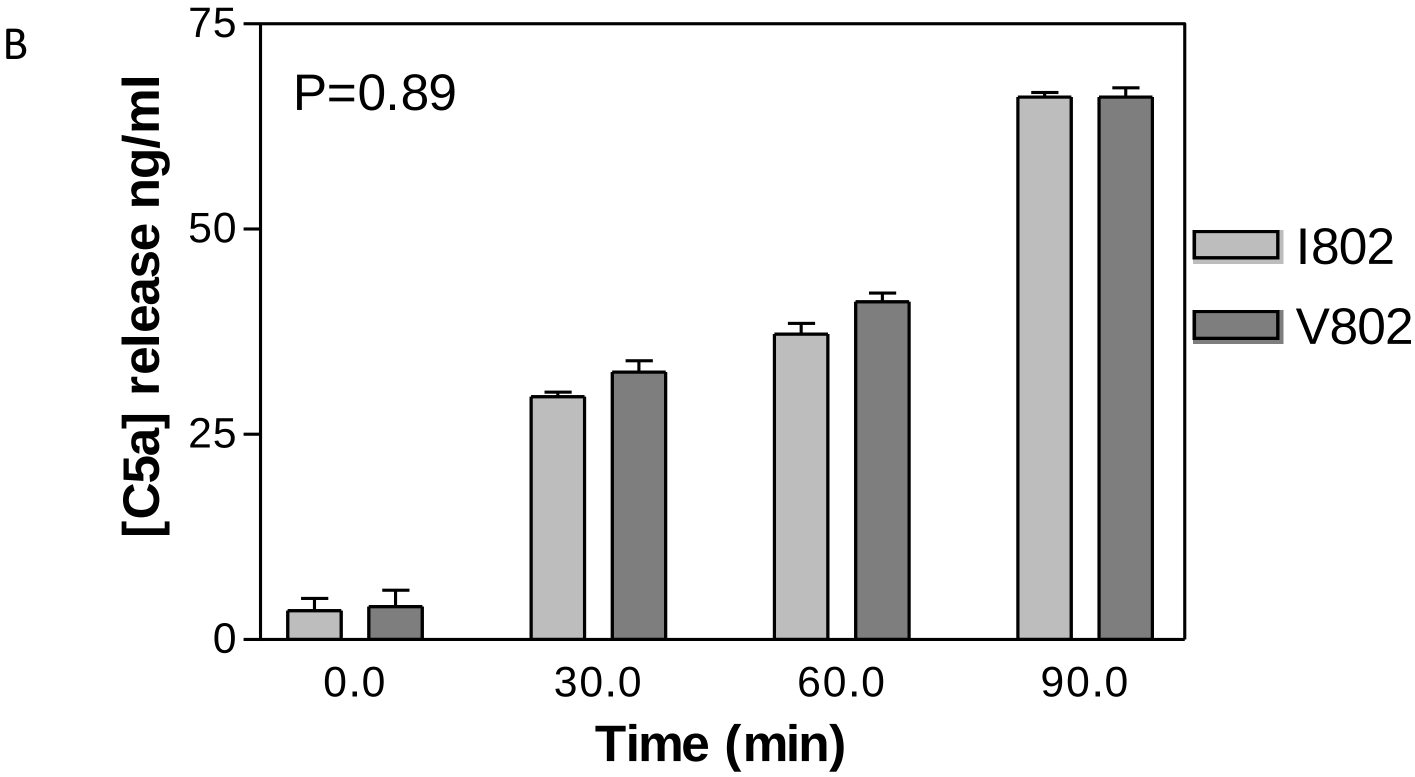

Figure S2. **Cobra Venom Factor assays show comparable cleavage of the two variants.**
A) Cleavage assays show that both C5 variants can be cleaved to C5α’ using CVFBb enzyme as analysed by SDS PAGE and quantified by densitometry (table), the rate of cleavage is comparable for both C5 variants. Gel shown is representative of three separate experiments.
B) C5a release was also measured in the samples at desired time points by ELISA. There was no significant difference in C5a generation observed.

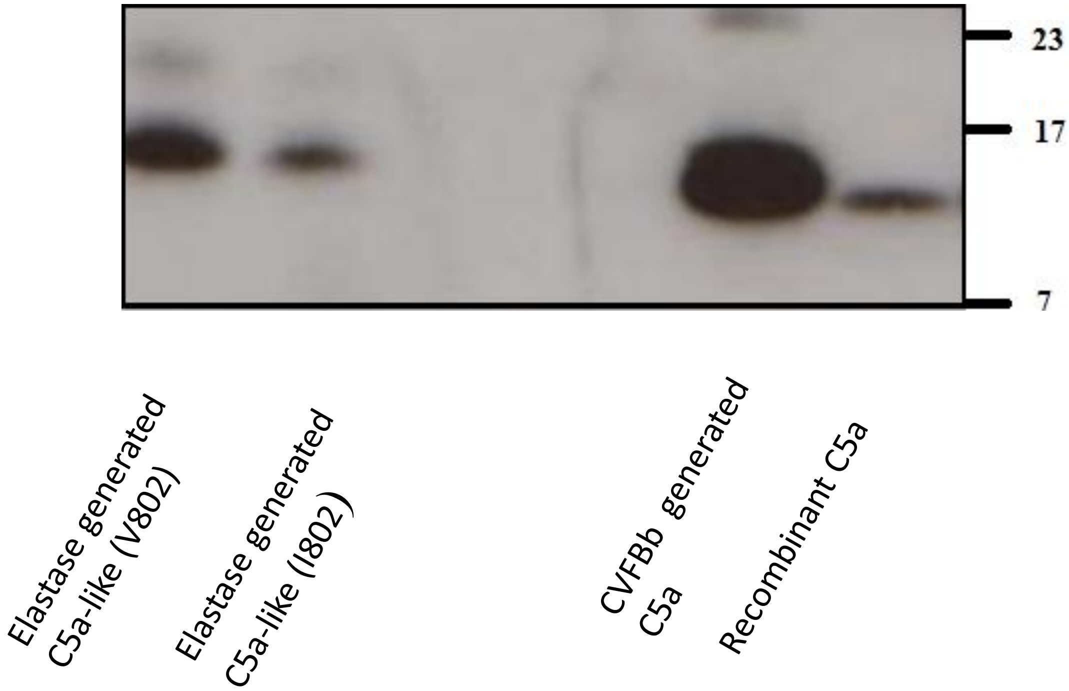

Figure S3. **C5a and C5 cleavage fragments sizes by western blot.**
C5a was generated by incubation of C5 variants with HNE or CVFBb for 1 hour at 37°C. Samples were run on SDS-PAGE, probed with anti-C5a and developed as a western blot. Recombinant C5a was also run as a control.
